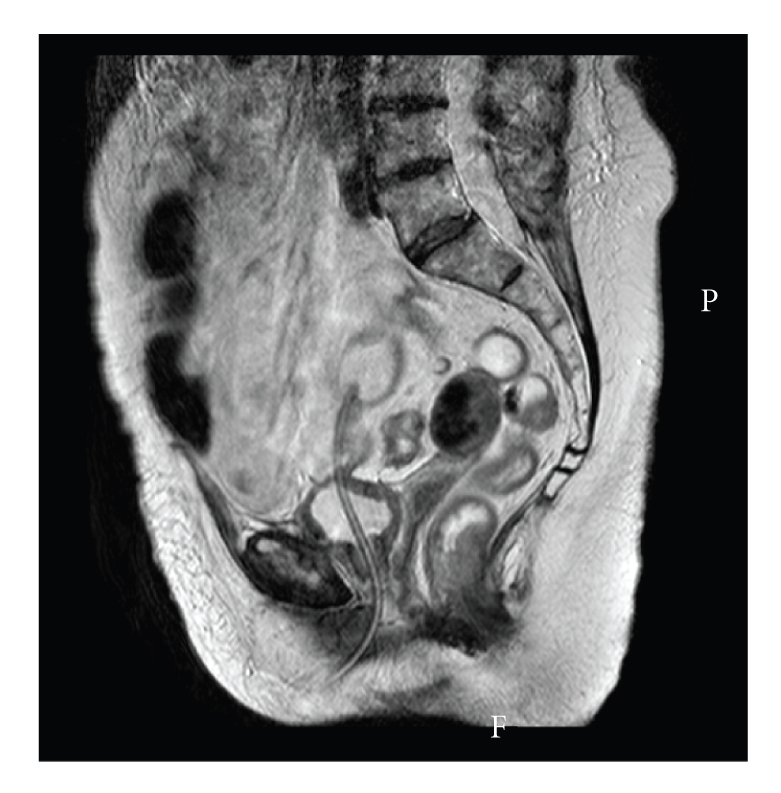# Enterovesical Fistula: A Rare Complication of Urethral Catheterization

**DOI:** 10.1155/2009/591204

**Published:** 2009-08-03

**Authors:** Amr Hawary, Laurence Clarke, Alasdair Taylor, Peter Duffy

**Affiliations:** Urology Department, Royal Lancaster Infirmary, Ashton Road, Lancaster, LA1 4RP, UK

## Abstract

This report describes the case of an eighty-two-year old lady with an indwelling urethral catheter inserted eight years prior to her presentation to manage her urinary incontinence. She underwent radiotherapy for muscle-invasive bladder cancer (stage T2b) in 1991 and had a laparotomy and drainage of an appendicular abscess in her early twenties. She presented with a short history of fecaluria, pneumaturia, and passage of urine per rectum. On laparotomy she was found to have an inflated catheter balloon that has eroded through the bladder wall into the lumen of a terminal ileal segment. To our knowledge this is the first reported case in literature of a patient developing an enterovesical fistula as a result of a urethral catheter eroding through the bladder wall into the bowel lumen. There are numerous known complications of long-term urethral catheterization. They include recurrent urinary tract infections, recurrent pyelonephritis, sepsis, urethral stricture, blocked and retained catheters, among many other reported complications. This case describes an unusual presentation secondary to an even more unusual complication. This should be considered when handling patients with indwelling urethral catheters inserted in unhealthy bladders.

## 1. Introduction

The symptoms of pneumaturia, fecaluria and passage of urine per rectum are worrying signs to any urologist. These usually indicate a communication between the gastrointestinal and urinary tracts. There are many reasons for such a complication to happen, whether intentionally as in bowl incorporation into the bladder or ureter, as a postoperative complication or as a complication of diseases of either system. We report a rare case of enterovesical fistula secondary to migration of urethral catheter into an ileal lumen and our management of this rare case. 

## 2. Case Presentation

An eighty-two-year old female presented to the urology outpatient clinic with a short history of fecaluria, pneumaturia, and passage of urine per rectum. She had an indwelling urethral catheter inserted, because of the total urinary incontinence, for a period of over eight years. This catheter was last changed twelve weeks prior to her visit to the urology clinic by her district nurse. The patient had positive MSUs in the year prior to her admission demonstrating mixed growth of enteric organisms, but no prior febrile urinary tract infections were reported.

She received radical radiotherapy alone for a muscle-invasive bladder tumor (stage T2b) in 1991 and underwent abdominal exploration for drainage of an appendicular abscess when she was in her early twenties. The patient had a background history of cerebrovascular disease, hypertension and ischaemic heart disease. She was felt to be an unsuitable candidate for radical surgery for her invasive bladder cancer due to these comorbidities. The decision to manage her incontinence with an indwelling urethral catheter was made having discussed available treatment options with the patient. A long-term indwelling catheter was felt to be the most appropriate option for her given her other extensive comorbidities. She did not have urodynamic testing prior to urethral catheterization. The exchanges of the catheter were being carried out every three months in the community and there had been no reported difficulty with her previous catheter changes. The patient was not undergoing routine cystoscopic surveillance of her bladder. 

On physical examination she was generally unwell with low-grade fever and pallor. Her abdomen was soft, mildly tender with no palpable masses and no signs of peritonitis. Her urethral catheter drained feculent material mixed with urine.

**Figure 1:**
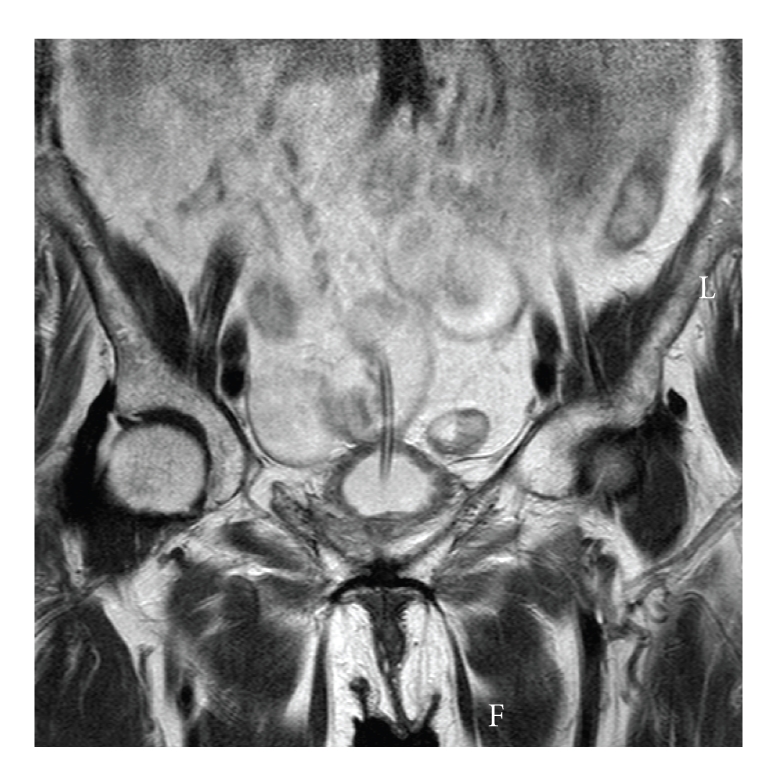


Laboratory investigations showed a low hemoglobin of 7.8 gm/dL, white cell count of 17 10^9^/L with a C reactive protein of 233. Magnetic resonance images (MRIs) of the abdomen and pelvis were performed urgently and were reviewed by a senior consultant radiologist and his impression was that those images confirmed the presence of the inflated balloon of the indwelling urethral catheter in the lumen of a bowel segment (Figures 1  and 2). 

In enterovesical fistulas treatment is undertaken depending on the aetiology, clinical status and general condition of the patient. Enterovesical fistulae seldom close spontaneously [1]. Given the nature of the pathology in this case and the presence of a foreign body in the fistulous tract (the urethral catheter), it was felt that the only way forward was to perform an urgent laparotomy and removal of the foreign body with excision of the fistulous tract. The situation was explained to the patient and she was consented for laparotomy, with all the risks and potential complications of surgery fully explained including both urinary and faecal diversion. On laparotomy there were severe adhesions, and the catheter balloon was found to have penetrated through the bladder wall and was lying in a terminal ileal segment adherent to bladder wall. Excision of this segment and urinary diversion by fashioning an ileal loop conduit was performed. A Hartmann's procedure was carried out due to the high clinical suspicion of presence of another separate colovesical fistula. The patient was transferred postoperatively to the intensive care unit and was moved afterward to the urology ward for 10 days. She had a smooth postoperative course with no recorded complications and is currently on regular urology outpatient clinic followup. Histology from the resected segment of terminal ileum demonstrated chronic inflammation only with no evidence of tumor recurrence.

## 3. Discussion

Enterovesical fistula was first described in literature by Cripps [2] with the pathognomonic features of fecaluria, pneumaturia and recurrent urinary tract infection. It is a rare but severe complication of pelvic radiation and it can occur in the absence of tumor recurrence [3]. Surgical procedures that resect the necrotic fistulized bowel and result in complete separation of the gastrointestinal and genitourinary tracts provide the best results in patients with radiation-induced enterovesical fistula. However, this is usually a very major procedure and urinary diversion may be necessary [4]. Enterovesical fistulas are relatively uncommon complications of colorectal and pelvic malignancies, diverticulitis, inflammatory bowel disease, radiotherapy and trauma. The major cause of this entity is malignancy, followed by diverticulitis, especially sigmoid diverticulitis [1]. In this case there was a history of both radiotherapy for invasive bladder tumor and drainage of appendicular abscess which did put her in a high-risk category for developing an enterovesical fistula. There is no consensus in literature as to what is the best surgical approach or technique to be implemented.

Many factors do come into the equation when the surgical approach is planned with the most important being patient's age, associated comorbidities, the nature of the fistula and the presence or absence of a foreign body.

The majority of complications associated with radical external beam radiotherapy for muscle-invasive bladder cancer can be subdivided into urinary and gastrointestinal complications which vary greatly in severity [5]. Urinary symptoms range from minor lower urinary tract symptoms such as frequency, dysuria, urinary retention and intermittent haematuria to more severe complications such as major haematuria, vesico-ileal/colic fistulae and bladder perforation. Gastointestinal complications include gastrointestinal obstruction secondary to strictures, lower GI bleeds, proctitis and diarrhoea. Every effort should be made to avoid the use of indwelling catheters in patients who have previously undergone pelvic radiotherapy in order to minimize the risk of catheter erosion through the bladder wall. In cases such as this, where patient comorbidities limit treatment options, consideration should be given to regular cystoscopic surveillance of the bladder in an attempt to anticipate and prevent such catheter-related complications.

## 4. Conclusion

This unusual case report describes one more complication of indwelling urethral catheters, which is to be added to a large and increasing list of complications.

It reasserts the fact that patients with unhealthy bladders are more prone to complications when left with an indwelling urethral catheter and raises the question of the need of this subgroup of patients to be kept under urological care rather than transferring them to the community.

## Figures and Tables

**Figure 2:**